# Comparison of inductively coupled plasma mass spectrometry and molybdenum blue colorimetry for total phosphorus determination in freshwater invertebrates

**DOI:** 10.1371/journal.pone.0317871

**Published:** 2025-01-28

**Authors:** Molly S. Costanza-Robinson, Baker J. Angstman, Qiting Cai, Charles Forbes, Julia S. Keon, Shuyi Lin, Emma D. Neill, Elizabeth G. Peebles, Ella Roelofs, Eric K. Moody

**Affiliations:** 1 Program for Environmental Studies, Middlebury College, Middlebury, Vermont, United States of America; 2 Department of Chemistry & Biochemistry, Middlebury College, Middlebury, Vermont, United States of America; 3 Department of Biology, Middlebury College, Middlebury, Vermont, United States of America; Albany Museum, SOUTH AFRICA

## Abstract

Molybdenum blue colorimetry (MBC) is the dominant, well-established method used for determining total P in environmental media, including in organismal tissues. However, other elemental methods for P determination are available, including inductively coupled plasma mass spectrometry (ICP-MS). Given the extensive literature using MBC to determine P in organismal samples, it is important to assess P analyses by ICP-MS and MBC to ensure that the two methods produce comparable data. In this work, we compared ICP-MS and MBC for total P determination in freshwater invertebrates, including the potential for analytical interferences, by applying both methods to three standard reference materials (SRMs) and 106 freshwater invertebrate samples. Average total P recoveries for SRMs were slightly higher for ICP-MS (99.8 ± 5.2%) than MBC (96.5 ± 5.4%), but both methods indicated good accuracy. Total P in invertebrates determined using the two methods was strongly linearly correlated (*r* = 0.96) with a slope of 1.01. On the whole, total P measured using ICP-MS exceeded that measured by MBC, but average pair-wise differences in %P were biologically negligible (0.044 ± 0.054). %P for SRMs and invertebrate samples run on ICP-MS in kinetic energy discrimination and standard modes compared favorably (e.g., SRM P recovery of 102% by both methods), indicating negligible influence of polyatomic ions on ICP-MS analysis. Similarly, analysis of P spike recoveries by ICP-MS (100.2 ± 3.4%) and MBC (107.0 ± 2.8%) were both considered acceptable. We conclude that ICP-MS represents a reliable and comparable alternative to MBC for determining total P in freshwater invertebrates while also offering the opportunity to measure additional biologically relevant elements in a single analysis.

## Introduction

Phosphorus (P) is an essential element that can limit biological growth, yet excess P runoff can lead to eutrophication of ecosystems and excess dietary P can have adverse impacts on the health and fitness of a variety of organisms [1–3]. For example, zooplankton in the genus *Daphnia* frequently exhibit reduced growth rates when feeding on diets high in P relative to other nutrients because excess P appears to cause metabolic shift that limit carbon uptake [3]. These effects are frequently studied in the context of ecological stoichiometry [4–6], which relies on ratios of available and constituent nutrients relative to P (e.g., C:P, N:P) in order to understand nutrient cycling [7–9], organismal traits and community dynamics [10–13], and longer-term ecological and evolutionary consequences of ecosystem change [14–18]. P generally makes up a small fraction of most biological tissues, however, with many organisms having less than 1% P by dry mass and even the most P-rich organisms rarely exceeding 6% P by dry mass [19–21]. Accurate and precise determination of total P within biota and other environmental media is therefore essential for investigating variation in ecosystem structure and function, as well as the degree of ecological impacts caused by eutrophication, climate change, and other anthropogenic ecosystem alterations.

Numerous methods have been proposed to quantify P in environmental media [22]. Of these approaches, molybdenum blue colorimetry (MBC) following acid digestion has been the dominant method since it was first proposed in 1962 [23–27]. MBC has been used for total P determination in soils [25, 28–30], sediments [31], algae and plants [32–34], and environmental waters [17, 18, 23, 25, 35–39]. MBC is quite nearly the sole method used for determining total P in animal tissues, including aquatic invertebrates [13, 33, 36, 37, 40–53].

In the field of ecology, an increasingly popular alternative approach to quantify P is inductively coupled plasma optical (or atomic) emission spectrometry (ICP-OES/AES). For example, ICP-OES has been used for P determination in fish and plant material [54–57] and in invertebrates [45]. When ICP-OES was compared to MBC for analysis of P in biomass, ICP-OES yielded lower body %P estimates in fishes but comparable estimates in other types of samples [58]. The digestion methods used for the MBC and ICP-OES samples differed, however, which may have contributed to the apparent differences by technique, particularly for recalcitrant fish tissues, such as bones and scales [58]. Investigation of alternative protocols and more direct comparison using uniform digestion methods will yield further insights into whether multi-element approaches can be used to reliably quantify P and other elements in biological samples.

Inductively coupled plasma mass spectrometry (ICP-MS), the focus of the current work, is another alternative to MBC that has provided reliable total P measurement in environmental waters [59], soils and sediments [29, 60–62], and plants [63–66]. A small number of studies have used ICP-MS to determine total P in invertebrates [46, 67], but a systematic evaluation of ICP-MS performance for this application and a direct comparison against the conventional MBC analysis is lacking. Indeed, some studies have used ICP-MS to determine other elements of biological interest (e.g., As, Fe, Zn, Cu, Mn, and base cations) and while also using MBC for P measurement [34, 68], suggesting that ICP-MS for P determination may not be understood to produce reliable data comparable to conventional MBC methods.

The underlying principles of MBC and ICP-MS differ considerably, which may give rise to differences in the fraction of P detected and the nature and magnitude of potential interferences. MBC relies on the selective complexation of specific P species (orthophosphate) to generate the blue color intensity that is measured using a spectrophotometer; thus, measurement of total P by MBC requires not only complete dissolution of P from organismal tissues but also its conversion to the orthophosphate form. In contrast, the ICP-MS plasma should non-selectively convert all forms of P in solution into the P-31 atomic ion measured by the mass spectrometer. Both methods are potentially susceptible to interferences. In MBC, high concentrations of As, F^-^, Si, Cr, NO_2_^-^, NO_3_^-^, S^-^, and oxidizing agents may lead to overestimates of P [35, 69–71], whereas high levels of Fe can lead to underestimates [72]. These interferences can vary in time due to their complex interactions with the development of the molybdenum blue complex [70]. In ICP-MS, the analysis of P is free from isobaric interferences, because it is the only element with an isotope of mass 31. However, formation of polyatomic ions with the same mass as P-31 (e.g., ^14^N^16^OH^+^, ^15^N^16^O^+^) can lead to overestimates of P [63, 73]. Additionally, the presence of other elements, particularly at high concentrations, in the matrix can influence ion formation and behavior in the plasma and downstream within the instrument to either elevate or suppress the signal from P [74]. Interferences for both MBC and ICP-MS are reduced by sample dilution and matrix-matching the calibration standards and unknowns [70, 74]. Interferences in ICP-MS can additionally be reduced by using internal standard calibration or the method of standard additions and by employing kinetic energy discrimination (KED) mode on the ICP-MS, which suppresses the signal from polyatomic ion interferences [67, 74, 75].

Because the use of ICP-MS for P determination offers promising potential to measure both P and other elements of biological interest [46, 67, 76], a direct comparison of P determination in organismal samples by both MBC and ICP-MS is needed. In this work, we determined total P in freshwater invertebrates using MBC and ICP-MS for split acid digests of standard reference materials and invertebrate samples obtained through the National Ecological Observatory Network (NEON). The two analytical approaches were compared and evaluated, including for total P recovery from standard reference materials (SRMs) and for method agreement when applied to freshwater invertebrates. The presence and magnitude of analytical interferences for an invertebrate matrix were evaluated using matrix spike recoveries and comparison of ICP-MS %P values when run in standard (STD) and KED modes.

## Materials and procedures

### Samples and standard reference materials

Invertebrates were collected in 2021 and 2022 from 14 freshwater sites in the United States following standard National Ecological Observatory Network (NEON) protocols, with the exception that samples were frozen rather than being preserved in ethanol [77]. Invertebrates were collected within Yellowstone National Park under National Park Service permit #YELL-2021-SCI-8186. All other invertebrate samples were collected by NEON staff and provided to the authors under assignable asset number 2020–514; any permits needed were issued to NEON directly. NEON samples were received and kept frozen prior to analysis. For analysis, NEON samples were thawed and organisms were identified to the lowest practical taxonomic level (frequently genus). This resulted in 106 discrete invertebrate samples (Table 1). Three standard reference materials were used to evaluate method accuracy (Table 2).

**Table 1:** Freshwater invertebrates obtained from 14 NEON sites and used to evaluate the comparability of using ICP-MS and MBC to determine total %P (n = 106).

Class	Order	Family	Count
Bivalvia	Venerida	Cyrenidae	1
Bivalvia	Venerida	Pisidiidae	1
Clitellata	Enchytraeida	Enchytraeidae	2
Clitellata	Rhynchobdellida	Glossiphoniidae	1
Gastropoda	Basommatophora	Planorbidae	2			
Gastropoda	Neotaenioglossa	Hydrobiidae	1			
Insecta	Coleoptera	Elmidae	16			
Insecta	Coleotera	Haliplidae	1
Insecta	Coleoptera	Hydrophilidae	1
Insecta	Diptera	Chironomidae	7
Insecta	Diptera	Empididae	1
Insecta	Diptera	Limoniidae	5
Insecta	Ephemeroptera	Baetidae	9
Insecta	Ephemeroptera	Caenidae	3
Insecta	Ephemeroptera	Ephemerellidae	6
Insecta	Ephemeroptera	Heptageniidae	7
Insecta	Ephemeroptera	Leptophlebiidae	1
Insecta	Hemiptera	Corixidae	5
Insecta	Lepidoptera	Crambidae	9
Insecta	Odonata	Coenagrionidae	2
Insecta	Odonata	Corduliidae	2
Insecta	Plecoptera	Chloroperlidae	2
Insecta	Plecoptera	Perlidae	6
Insecta	Plecoptera	Perlodidae	1
Insecta	Trichoptera	Brachycentridae	1
Insecta	Trichoptera	Hydrobiosidae	1
Insecta	Trichoptera	Hydropsychidae	1
Insecta	Trichoptera	Limnephilidae	4
Insecta	Trichoptera	Polycentropodidae	1
Insecta	Trichoptera	Rhyacophilidae	4
Malacostraca	Amphipoda	Gammaridae	1
Malacostraca	Amphipoda	Hyallelidae	1

**Table 2:** Total phosphorus as percent dry mass for the three standard reference materials (SRMs) used to evaluate method accuracy.

SRM	Certifier	Total P ± SE (% Dry Mass)	Certification level^b^
1570a Spinach Leaves	USA NIST	0.5187 ± 0.0067	Certified
SQID-1 Cuttlefish	Canada CNRC	0.8400 ± 0.0400	Reference
CRM No. 15 Scallop	Japan NIES	0.9560^a^	Reference

^**a**^ no statement of uncertainty in %P was provided

^b^ certified values are the result of more comprehensive testing and reporting than reference values, and typically have tighter tolerances.

### Chemicals and reagent preparation

Trace-metal grade sulfuric, hydrochloric, and nitric acids were purchased from Fisher Scientific (Waltham, MA, USA). For colorimetry, ammonium molybdate was purchased from Sigma-Aldrich (St. Louis, MO, USA). L-(+)-ascorbic acid was purchased from Merck (Darmstadt, Germany). All reagents were of analytical grade.

All glassware was acid-washed prior to use, and water was purified (Hydro Services Picotech 2, Durham, USA). An acid molybdate solution containing H_2_SO_4_ (1.62 M), ammonium molybdate (6.07 mM), and antimony potassium tartrate (2.10×10^−4^ M) was prepared in water and stored in an amber glass bottle for up to 6 months at 4°C. Ascorbic acid solution (0.14 M) was prepared fresh daily. Color reagent for MBC was prepared as a 1:4 mixture of ascorbic acid and acid molybdate.

MBC calibration standards were made from a 1000 μg/mL P commercial stock (Inorganic Ventures, Christiansburg, USA). Calibration standards for ICP-MS were prepared by diluting a custom-mixed stock (71A and 71B, Inorganic Ventures) containing P and 47 other elements and spiking with an internal standard solution made from 10 ppm Sc, Y, and Bi single-element solutions (Inorganic Ventures).

### Sample preparation and digestion

For larger invertebrates, individuals were analyzed as a whole individual sample or as a homogenized subsample of a single individual. When available and necessary to achieve adequate sample mass, multiple individuals of the same genus from the same NEON sample were pooled into a single sample. Following identification, invertebrate samples were dried at 60°C for at least 48 h. SRMs were dried according to methods specified on their certificates. Samples (0.75–5 mg for invertebrates; 3–5 mg for SRMs) were combusted in glass vials in a muffle furnace at 550°C for 4 h.

To the glass vial containing ashed invertebrate or SRM, 2 mL of 1 M HCl and 10 mL of ultrapure water were added. The vials were capped and samples digested in an oven at 100°C for 3 h. Digests were cooled to room temperature and filtered (0.22 μm nylon). Filtered digests were split for subsequent preparation for MBC or ICP-MS. Method blanks and SRMs were prepared with each digestion batch in the same manner as invertebrate samples. Sample preparation and digestion methods were generally consistent with methods outlined in established methods guides [22], with the following modifications. First, instead of using biotic tissue SRM digests to calibrate the instruments, we used matrix-matched aqueous standards for calibration, analyzed SRMs as unknowns, and evaluated method accuracy by calculating SRM recoveries. Second, we avoided sample transfers by weighing dried invertebrate samples directly into the digestion vessels, which substantially improved SRM recoveries (data not shown). Third, we filtered samples, which is required by our institutional policy for the shared-used ICP-MS; clean method blanks and good SRM recoveries demonstrated that filtering did not compromise the analyses. Finally, our dilution factors were higher than suggested by Benstead et al., [22] because we needed larger sample volume to accommodate analysis by two methods. An implication of our higher dilutions is that the sensitivities of our ICP-MS and MBC methods are inferior to what would be achieved when performing either method alone. Because of this, analytical sensitivity is not a focus of the current method evaluation and comparison.

### Molybdenum blue colorimetry (MBC)

MBC methods were adapted from Murphy and Riley [20]. Aliquots of filtered acid digests were diluted 5-fold with water and mixed 5:1 with the color reagent. This resulted in a 6-fold dilution of the MBC split acid digests prior to analysis. Calibration standards were matrix-matched to samples with respect to HCl and mixed with color reagent in the same ratio as samples. Samples and standards were vortexed and allowed to sit at least 15 min and no longer than 30 min for color development. Spectrophotometry (8453 G1103A, Agilent Technologies, Palo Alto, USA) was performed at 885 nm.

External calibration (blank-corrected) was initially performed between 1 and 2000 μg/L using 1, 5, and 10-cm pathlength glass cells. The linear quantification range was defined by the lowest concentration standard that fell within 15% of the calibration line and had relative standard deviation <15% for triplicate measurements. The upper end of the linear range was defined as the highest concentration standard that met these same quality control criteria and allowed adequate linearity (r^2^ > 0.99). The 1-cm cell was used for remaining MBC analyses, because it afforded a large linear quantification range, had sufficient sensitivity for nearly all samples, and required lower sample volume. Method blanks were analyzed with each sample batch and were below quantification for P. To evaluate the potential influence of matrix effects, a P-spike recovery test was performed on 10 invertebrate samples and 9 replicates of the spinach SRM. The amount of P spiked was specific to the sample to ensure that spiked digests remained within the linear quantification range and represented an increase in P concentration of 100–300%.

### Inductively coupled plasma mass spectrometry

To matrix-match the samples and calibration standards with respect to HNO_3_ and HCl and to reduce the potential for Cl^-^ interferences, aliquots of the filtered digests were diluted 5-fold using 0.5% HNO_3_. The ICP-MS (iCAP-Q, Thermo Fisher Scientific) was equipped with an ASX-520 autosampler, PFA-ST microflow nebulizer, and quartz cyclonic spray chamber. A standard quartz torch and injector (2.5 mm inner diameter), nickel sample cone (Analytical West) and skimmer cone (high matrix, Thermo Fisher) were used. The instrument was tuned to reduce formation of oxides and doubly charged ions using a tune solution (Thermo-4AREV, Inorganic Ventures). All method blanks, calibration and quality control standards, unknowns, and SRMs were spiked with an internal standard solution to achieve 5 μg/L Sc-45 [63].

Internal standard calibration (blank-corrected) was initially performed from 1 to 2000 μg/L P-31. The linear quantification range was defined by the lowest standard that fell within 20% of the calibration line, had relative standard deviation <20%, and exhibited linearity (r^2^ > 0.99). Calibration linearity extended to the highest concentration standard run, and therefore the upper end of the linear range was not determined. Internal standard counts remained stable within 15% over the course of the run. Method blanks were below quantification limits for P, and calibration accuracy was verified to be within 15% using an independently prepared 300-μg/L P quality control standard, run after every group of 10 samples. All method blanks, calibration and quality control standards, unknowns, and SRMs were analyzed in triplicate (%RSD within 15%). P-spike recovery tests were performed on 10 invertebrate samples and 9 replicates of the spinach SRM to evaluate the potential for matrix interferences, as described for MBC.

ICP-MS was run in KED mode for all samples to reduce the potential for polyatomic ion interferences; however, 24 invertebrate samples and 12 replicates of the spinach SRM were additionally analyzed in STD mode, specifically to evaluate the potential magnitude of polyatomic interferences. Polyatomic ions that have the same mass as P-31 (e.g., ^14^N^16^OH^+^, ^15^N^16^O^+^) can be formed within the plasma and passed on to the mass spectrometer, artificially elevating the apparent amount of P-31 in the sample [63, 73]. KED mode relies on a collision cell in which gas atoms (He, in our case) collide disproportionately with larger cross-section ions (e.g., polyatomic ions), which reduces their kinetic energy and precludes them from being passed along to the mass spectrometer for detection and quantification. The result of running in KED mode is a decrease in overall analytical sensitivity but also a relative enhancement of the signal of the desired monatomic (e.g., P-31) ion over polyatomic ions of the same nominal mass.

### Quality control and data analysis

Following verification of normality and equal variances using Shapiro-Wilk and F tests, respectively, a two-way ANOVA was used to examine differences among groups, including between SRM total P recoveries by analytical method and SRM type. If normality was not achieved, a Wilcoxon signed-rank test was used for comparisons, including for pair-wise comparison of the total P fraction in NEON samples measured by MBC versus ICP-MS. For all tests, significance was taken as *p* < 0.05.

## Results

### Calibration and linear quantification range

**Fig 1:**
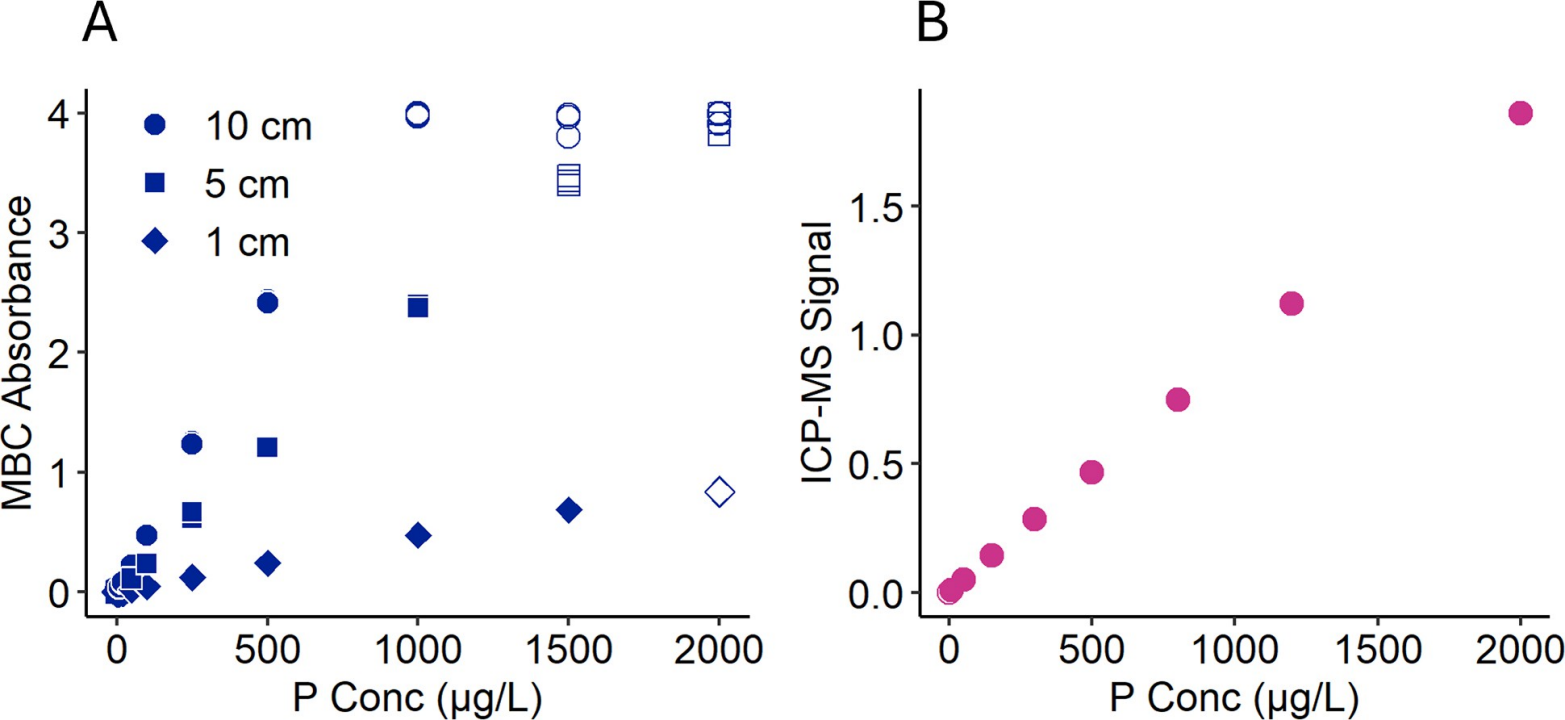
Calibration curves for molybdenum blue colorimetry (MBC) and inductively coupled plasma mass spectrometry (ICP-MS). A) Calibration curves for MBC were measured using 1-, 5-, and 10-cm pathlength cells, with signal (y-axis) reported as blank-corrected Absorbance (885 nm). B) Calibration curves for ICP-MS (right) were measured in KED mode with the signal reported as the ratio of blank-corrected counts-per-second (cps) for *m*/*z* 31 (P) and 45 (Sc, internal standard). In both plots, filled markers indicate points that satisfied quality control criteria and were considered to be within the linear quantification range.

Representative calibration curves for MBC (1-, 5-, and 10-cm pathlength cells) and ICP-MS (KED mode) are shown in Fig 1 (zoom to low concentration shown in S1 Fig). Filled markers indicate the calibration standards that met the quality control criteria and were considered to be within the linear quantifiable range. ICP-MS calibration curves were linear out to the highest calibration standard concentration tested, and therefore, these data do not capture the true upper limit of linearity. MBC calibration curves plotted using absorbance normalized by pathlength collapsed into a single line, as expected (S2 Fig). The linear quantification ranges determined for our methods are shown in Table 3.

**Table 3:** Linear quantification range for the molybdenum blue colorimetry (MBC) and inductively coupled plasma mass spectrometry (ICP-MS) kinetic energy discrimination (KED) methods used.

Method	Linear Quantification Range (μg/L)
**ICP-MS**	10–2000^a^
**MBC**	
1-cm	100–1500
5-cm	25–1000
10-cm	10–500

^a^ ICP was linear through 2000 μg/L, the highest concentration standard tested; linearity may extend to higher concentrations.

### Total P in SRMs by MBC and ICP-MS

**Fig 2:**
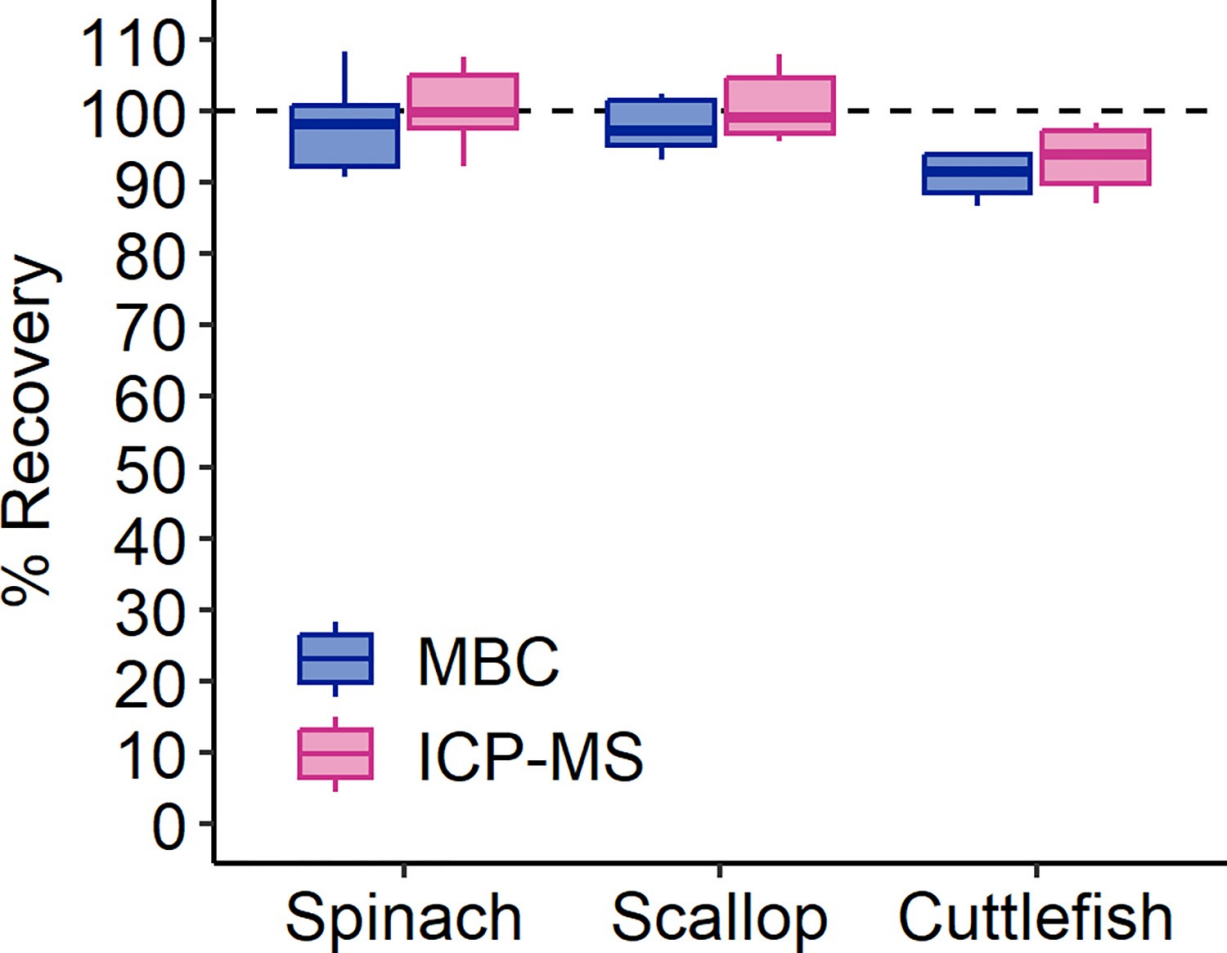
Total P recoveries for standard reference materials by MBC and ICP-MS. P recoveries (percent) in split digests of scallop (*n* = 6), spinach (*n* = 17 (MBC); *n* = 20 (ICP-MS)), and cuttlefish (*n* = 4) reference materials (see Table 2). The dashed line represents 100% P recovery. P recovery was higher for ICP-MS (*F*_1,53_ = 6.9, *p* = 0.011) and differed by SRM (*F*_2,53_ = 7.6, *p* = 0.0013).

Method blanks, run with each digestion batch, contained P below the quantification limit for both MBC and ICP-MS, indicating negligible sample contamination. To examine the accuracy resulting from the baseline methods (e.g., 1-cm pathlength cells for MBC and KED mode for ICP-MS), total P was determined for replicates of three SRMs using both MBC and ICP-MS. Total P recoveries ranged from 86.7–108.3% and averaged 96.5 ± 5.4% and 99.8 ± 5.2% for MBC and ICP-MS, respectively (Fig 2). P recovery was higher for ICP-MS than for MBC (*F*_1,53_ = 6.9, *p* = 0.011) and differed by SRM (*F*_2,53_ = 7.6, *p* = 0.0013), with lower overall P recoveries by both methods from the cuttlefish SRM.

### Comparison of total P in invertebrates by MBC and ICP-MS

**Fig 3:**
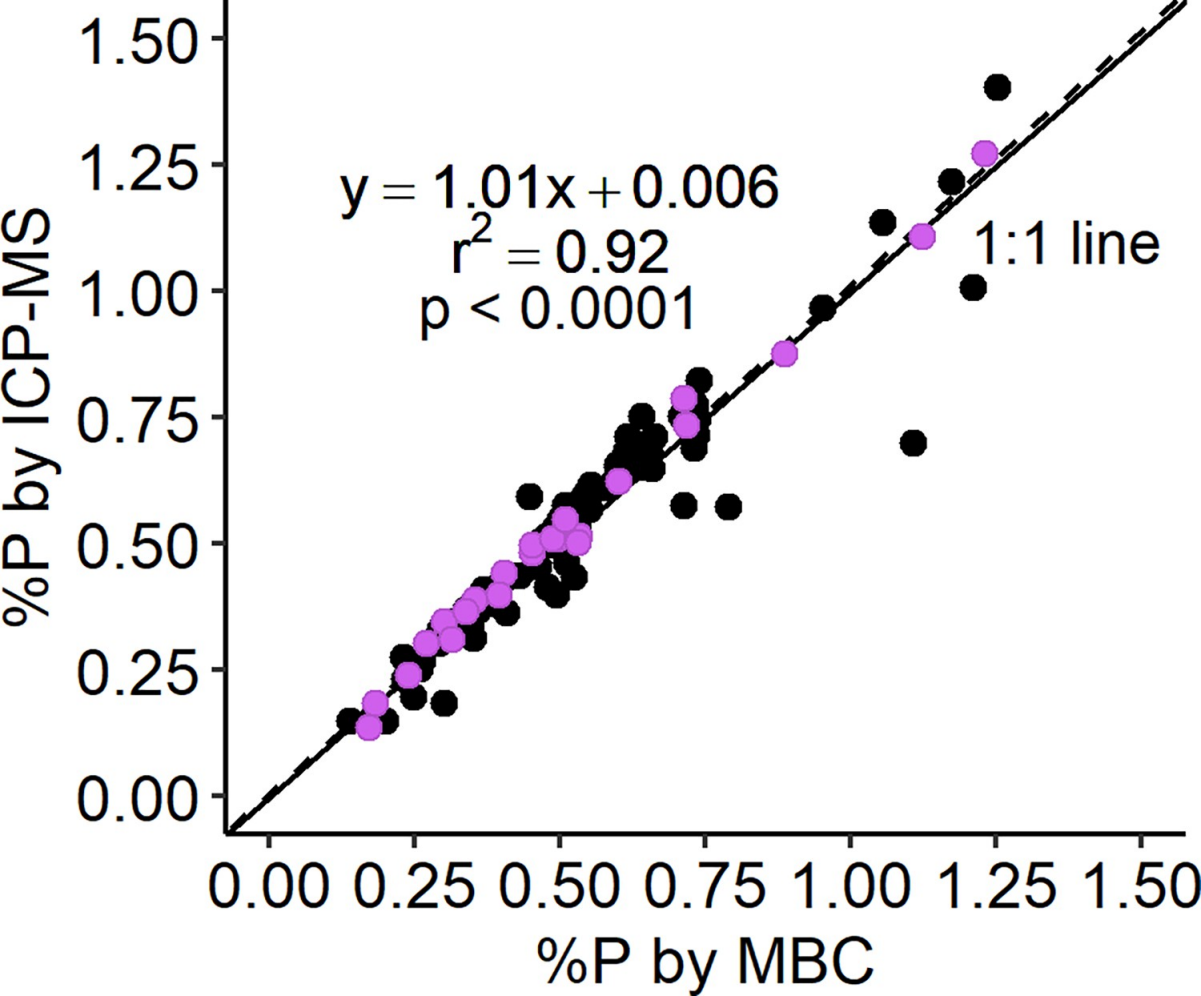
Total %P determined for freshwater invertebrates (*n* = 101) by MBC and ICP-MS. The dashed and solid lines represent the Deming regression (equation on plot) and 1:1 line, respectively. Purple markers indicate invertebrate samples that were additionally used to evaluate analytical interferences.

Total P in split digests of invertebrates (*n* = 106) were determined using MBC and ICP-MS, from which the %P was calculated (Fig 3). The diluted digests of 5 samples were below quantification by either or both ICP-MS or MBC, and therefore are not included in Fig 3 and related statistics. %P determined using the two methods was strongly linearly correlated (Pearson’s, *r*_*99*_ = 0.959, *p* < 0.0001) and closely tracked the 1:1 line (Deming, *slope* = 1.01). ICP-MS and MBC values differed (*W* = 1420, two-tailed *p* < 0.001), with ICP-MS generally higher as was observed for the SRMs (S3 Fig). The average pairwise absolute difference in invertebrate %P values was only 0.044 ± 0.054. The difference between methods amounted to an average measurement discrepancy of 8.3%. Larger discrepancies between methods were observed for a small number of samples.

### Polyatomic interferences and ICP-MS

**Fig 4:**
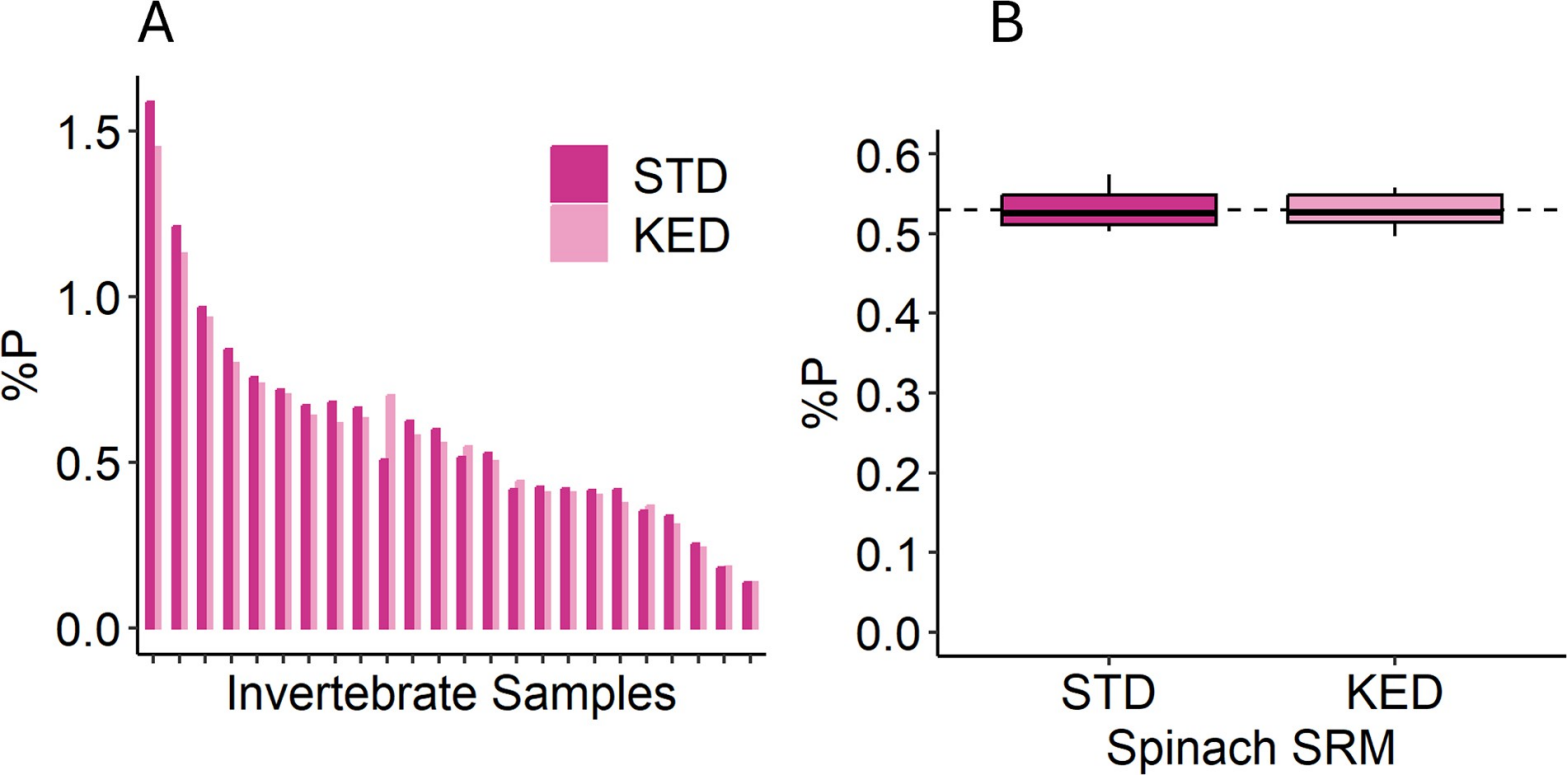
Comparison of ICP-MS %P measured using KED and STD modes. %P measured in A) invertebrates (n = 24) and B) spinach SRM (n = 12). The dashed line in the SRM plot shows the certified %P (0.52%). %P was higher when measured in STD mode than in KED mode (*W* = 159, two-tailed *p* = 0.0053), but the absolute pair-wise differences were small (0.056 ± 0.051%P).

%P determined using MBC and ICP-MS were in close agreement for the great majority of samples, even as more significant method-specific discrepancies were observed for a small number of samples. To evaluate the potential impact of polyatomic ions on ICP-MS determination of %P, digests of 24 NEON invertebrate samples (purple markers, Fig 3) and 12 replicates of spinach SRM were analyzed both in KED and STD modes. Overall, STD %P values exceeded those measured using KED (Fig 4, *W* = 159, two-tailed *p* = 0.0053), suggesting some influence of polyatomic ions on P determination when running in STD mode. The difference between STD and KED P determinations was biologically small (average absolute pair-wise difference in %P of 0.031 ± 0.038), however. SRM recoveries in STD and KED modes were 102.2 ± 4.5% and 102.0 ± 4.1%, respectively. In contrast to the small difference in %P by mode, the analytical sensitivity was far superior in STD mode. KED analytical signals were <1% of STD signals and associated with greater variability across triplicate measurements. As a result, the lower limit of quantification for KED mode was 10 ppb, while for STD mode, the lower limit of quantification decreased to 1 ppb, the lowest calibration standard run.

### Evaluating P matrix spike recoveries in MBC and ICP-MS

**Fig 5:**
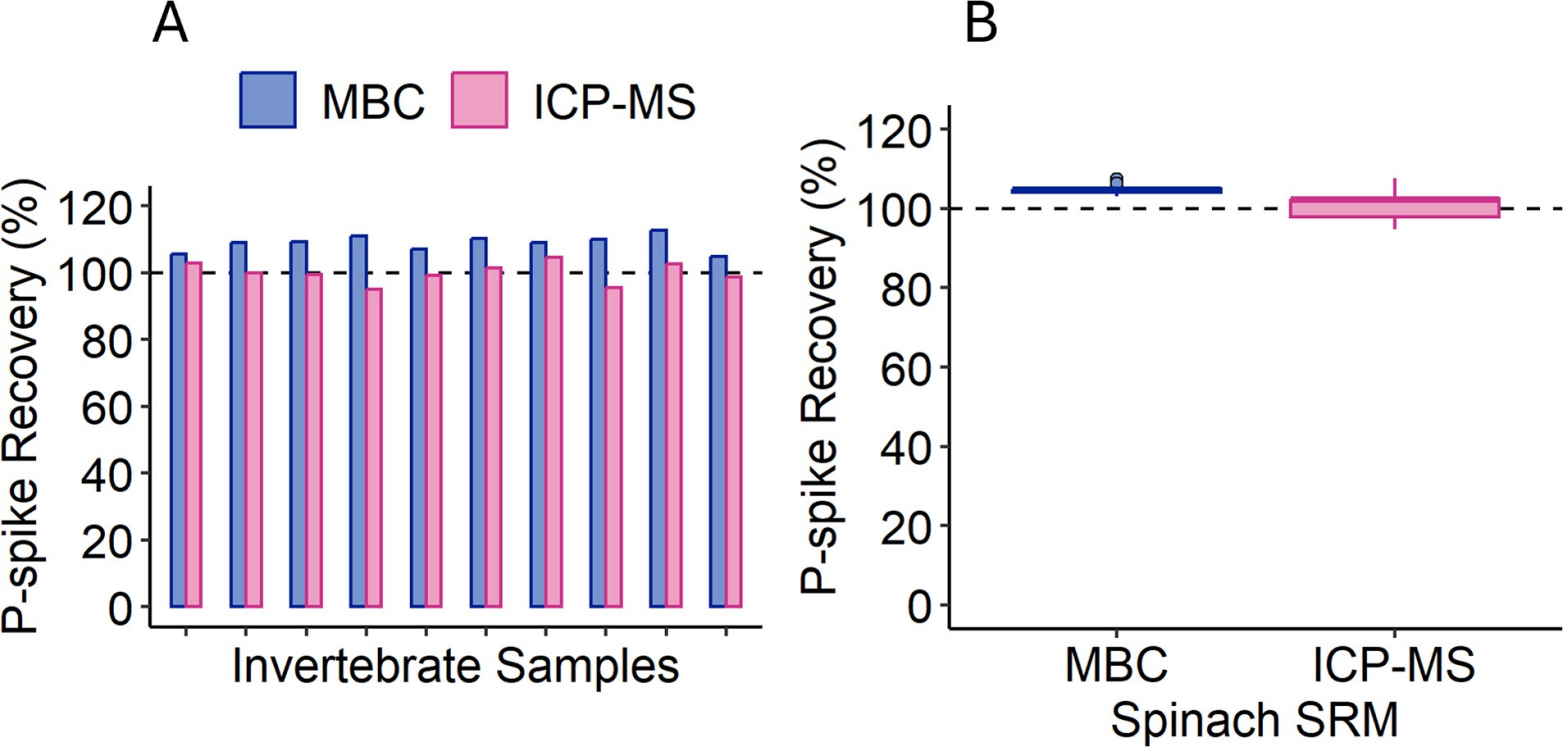
Comparison of P-spike recoveries for MBC and ICP-MS. Recoveries of P spikes for A) invertebrates (n = 10) and B) spinach SRM (n = 9). The dashed line shows 100% recovery of total P. Overall, %P values were higher than ICP-MS values (*t*_*18*_ = 6.3, two-tailed *p* < 0.0001).

Both ICP-MS and MBC are potentially subject to other types of interferences from the sample matrix, which can be revealed through analysis of analyte spike recoveries from the unknown sample matrix. In this work, digests of 10 invertebrate samples and 9 replicates of spinach SRM were additionally analyzed by MBC and ICP-MS after addition of known amounts of P (e.g., spike additions). Phosphorus spike recoveries were higher for MBC than ICP-MS (107.0 ± 2.8% and 100.2 ± 3.4%, respectively) (Fig 5, *t*_*18*_ = 6.3, two-tailed *p* < 0.0001), the opposite of the general trend in %P values measured for these invertebrate and spinach SRM samples when unspiked. Although the differences in the spike recoveries for MBC and ICP-MS were statistically significant, the spike recoveries measured for each method were considered to be well within the acceptable range.

## Discussion

Our results suggest that P concentration data obtained by ICP-MS are by and large comparable to those produced by molybdenum blue colorimetry. We found that P concentrations measured in samples using each method were strongly correlated with each other with a slope of 1.01, suggesting that the two methods consistently produced very similar results for a range of taxonomically diverse freshwater invertebrates. Further, limited evidence of polyatomic or matrix interferences in ICP-MS suggests this method can be used reliably for quantification of P in animal tissues.

Average total P recoveries in SRMs were 96.5 ± 5.4% and 99.8 ± 5.2% for MBC and ICP-MS, respectively (Fig 2). Analyte recovery from SRMs near 100% indicates the accuracy and reliability of both analytical methods, insofar as the SRM matrix adequately represents the sample matrix of interest. A freshwater invertebrate SRM closely related to our sample type was not found. We selected the NIST Spinach SRM because others have used it in invertebrate studies that rely on MBC (e.g., [78–80]) and because it has certified values for P content (as opposed to reference values). The spinach SRM recoveries in this work are similar to those reported for a peach leaf SRM by others (e.g., [16]). The scallop and cuttlefish SRMs are more closely related taxonomically and matrix-wise to freshwater invertebrate unknowns than the spinach but have drawbacks: the P content values provided for these SRMs are reference values and, for the scallop, do not include established uncertainties. While none of the SRMs provide an ideal indicator of overall method accuracy specifically for invertebrates, the good P recovery results for both MBC and ICP-MS for all three SRMs provide compelling evidence that sample digestion was sufficient and that ICP-MS and MBC can provide similarly reliable values for total P in biotic samples.

Total %P in invertebrates measured by ICP-MS and MBC were highly correlated and closely tracked a 1:1 line (Fig 3). Despite ICP-MS %P statistically exceeding MBC %P, average pair-wise differences between methods were only 0.044%P, which was considered biologically negligible as body %P varies from less than 0.1% to over 10% among aquatic organisms [81]. Still, the higher %P determined using ICP-MS for most invertebrate samples was consistent with the higher (and closer to 100%) ICP-MS recoveries of P from the SRMs. These small differences may point to slight underestimation of %P by the MBC methods we employed, potentially due to incomplete conversion of total P to the detectable orthophosphate form [24, 35, 76].

Although both methods recovered close to 100% of the SRM P and differences between methods were small, two approaches were used to understand the potential for matrix interferences. First, %P determination using ICP-MS KED and STD modes were compared to evaluate the potential for polyatomic interferences [74]. STD %P values were slightly elevated compared to KED %P values, indicating that polyatomic ions may elevate %P values somewhat if KED mode is not used. Even so, the average pair-wise differences were quite small (0.031%P on average), and average P recoveries from SRMs for both modes were 102%. These findings suggest that polyatomic ions are not likely to be of high concern for determining %P in invertebrates by ICP-MS and that both modes of ICP-MS produce reliable P determinations for these samples. While the use of KED reduces the probability of polyatomic ions artificially elevating values, which can be important when analyzing diverse unknown samples, it simultaneously decreases method sensitivity. In our case, running in KED mode as opposed to STD mode, but keeping all other method parameters unchanged, decreased the analytical signal more than 100-fold and increased the lower limit of quantification from 1 ppb, the lowest standard we ran, to 10 ppb. The loss of sensitivity due to running in KED mode only hindered the ICP-MS quantification of P in two samples of the 106 invertebrate samples we analyzed. These samples consisted of a hydrobiid snail analyzed with shell and an early instar limnephilid caddisfly, which both represent samples with low body mass and low body P concentrations. Given that our samples were diluted more than typical to accommodate the sample-volume needs of our methods comparison, we suggest that ICP-MS sensitivity is not a limiting factor and both KED and STD modes would be well-suited to analysis of P in invertebrates. The improved sensitivity of running ICP-MS in STD may be particularly useful in some situations, however.

Further supporting the argument that matrix effects had little, if any effects on either analysis, P spike recoveries for ICP-MS and MBC methods were 100.2 ± 3.4 and 107.0 ± 2.8%, respectively. Despite a host of potential matrix interferences that can compromise P determination, such as from As, Si, F, Fe, Ca, Mg, and Na for MBC [35, 69–71] and polyatomic ions and space-charge effects for ICP-MS [74], P-spike and SRM P recoveries showed little evidence of matrix effects. The lack of matrix effects for either analytical method could be a result of overall low concentrations of interfering chemical species in the organisms and/or to the dilution of sample digests used here [70, 74]. Best practice interference-reduction approaches employed, including use of matrix-matched calibration standards for both methods, the use of internal standards, and instrument tuning to minimize doubly charged ions for ICP-MS, may have also limited the impact of any matrix effects. As noted earlier, to accommodate the evaluation of multiple methods, our digest dilutions were higher than some others have used, which simultaneously reduces analytical sensitivity and the potential for analytical interferences. In our case, the reduced sensitivity hindered the quantification of P in a small number of invertebrate samples (<5%), suggesting that both MBC and ICP-MS are generally sensitive enough to run at dilutions that are robust to matrix interferences. If more concentrated samples are used, assessment of interferences and use of interference-reducing best practices would be recommended, although the smaller linear range of quantification for MBC might necessitate dilution of higher concentration samples regardless.

In summary, this study provides compelling evidence that ICP-MS is a reliable and comparable alternative to MBC for determining total P in freshwater invertebrates. ICP-MS and MBC delivered comparable total P recoveries from SRMs near 100% and comparable values of total %P from diverse invertebrate samples. Analytical interferences from polyatomic ions or other matrix effects were negligible, with both ICP-MS and MBC methods delivering comparable recoveries of matrix P-spikes near 100%. The comparability of the measurements and similar robustness to interferences suggests data from both methods can be readily compared and interpreted without methodological distinctions. Therefore, users of ICP-MS interested in acquiring data on multiple elements in organismal samples can proceed with confidence in using P data thus acquired. Further, we have no reason to believe that ICP-MS would not also provide MBC-comparable data for other types of organismal samples. Analytical measurements are subject to instrument-specific factors (e.g., instrument sensitivity, instrumental parameters, and maintenance) and to laboratory environmental conditions, however. Thus, regular instrument maintenance, optimization of system parameters, and proper calibration is essential for ensuring the reliability of data and its comparability across MBC and ICP-MS and across institutional settings.

Despite delivering comparable %P data, each method has potential advantages and disadvantages. The advantages of MBC are well-established and account for its enduring use [76]. MBC provides accurate and precise %P determination at relatively low cost and does not typically require extensive training to perform nor to maintain the associated equipment. The linear quantification range can be tailored by using different pathlength cells and is large enough to be convenient for a diverse set of samples. Downsides of MBC include the need to make up the reduction agent (e.g., ascorbic acid) fresh daily and the expiration of samples due to degradation of the blue complex within hours, in contrast to the shelf-stable reagents for ICP-MS. Advantages of ICP-MS analysis include its high sensitivity, particularly if ICP-MS is run in STD mode, which might be useful if invertebrate mass is limited or for analysis of low %P samples. The sensitivity and relatively large linear quantification of range of ICP-MS readily accommodate a large range in sample P concentrations, which might result from variable available organismal mass or organismal %P. ICP-MS can be used for multi-element analysis, allowing for dozens of elements to be measured simultaneously with little additional sample preparation or analysis time or expense. Examination of multi-elemental organismal composition can contribute to our understanding of relationships between resource availability and organism function and of basic features of homeostatic regulation [6, 67, 82]. It can also allow us to understand and predict the ecological consequences of nutrient addition or toxic pollution [9]. Digested ICP-MS samples also hold indefinitely due to acid preservation, which adds flexibility. Downsides to ICP-MS include its acquisition and maintenance costs, which may be cost-prohibitive for many institutions [76]. ICP-MS also requires more extensive training to use and to maintain than typical MBC equipment.

In summary, we provide evidence that ICP-MS is a reliable alternative to the conventional MBC method for determining total P in freshwater invertebrates. The two methods provided comparable and reliable P determinations, as evidenced by total P recoveries from SRMs near 100% and highly correlated and similar values of total %P from invertebrate samples. Both methods were robust to potential analytical interferences. Despite delivering comparable data, each method has advantages. ICP-MS exhibits greater analytical sensitivity, larger linear quantification range, and multi-element capability, but at higher cost and with more extensive training required. The shelf-stable reagents used in ICP-MS and the setup of an instrument with an autoanalyzer could improve efficiency and throughput over MBC, but this will greatly depend on the particular setup of the instrument in a given laboratory. MBC is a highly reliable, simpler method that is more accessible from a cost, maintenance, and training standpoint.

## Supporting information

S1 Fig Low concentration region (<500 ppb) of calibration curves for molybdenum blue colorimetry and inductively couple plasma mass spectrometry. (TIF)

S2 Fig Pathlength-normalized calibration curve for molybdenum blue colorimetry. (TIF)

S3 Fig Pair-wise differences in %P measured using inductively couple plasma mass spectrometry and molybdenum blue colorimetry. (TIF)
