# Supplementary figures and images for: Comparison of inductively coupled plasma mass spectrometry and molybdenum blue colorimetry for total phosphorus determination in freshwater invertebrates
Source: PLoS One. 2025 Jan 28;20(1):e0317871. doi: 10.1371/journal.pone.0317871 (PMC11774397; doi:10.1371/journal.pone.0317871)

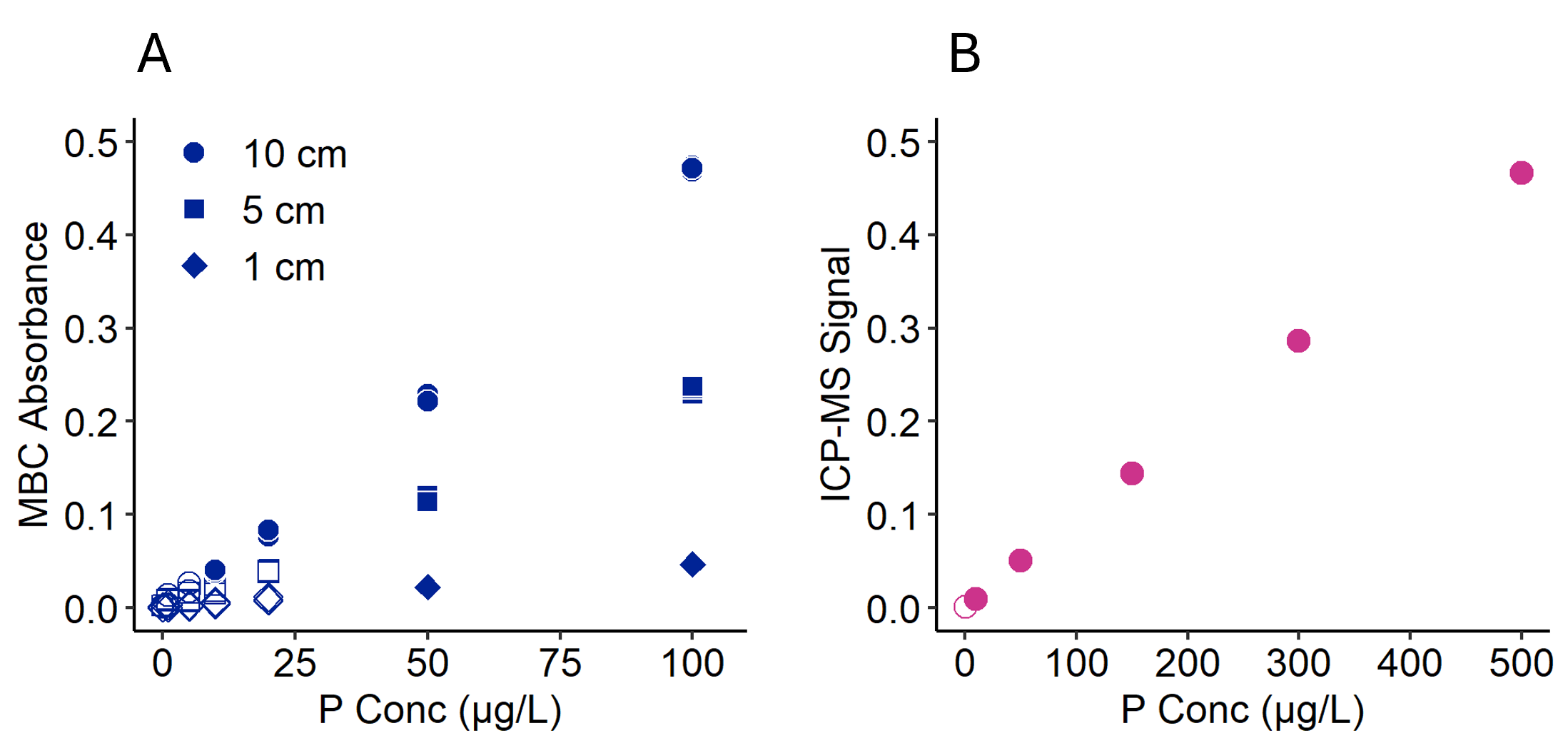

Supplement: S1 Fig — (TIF) [file pone.0317871.s001.tif]

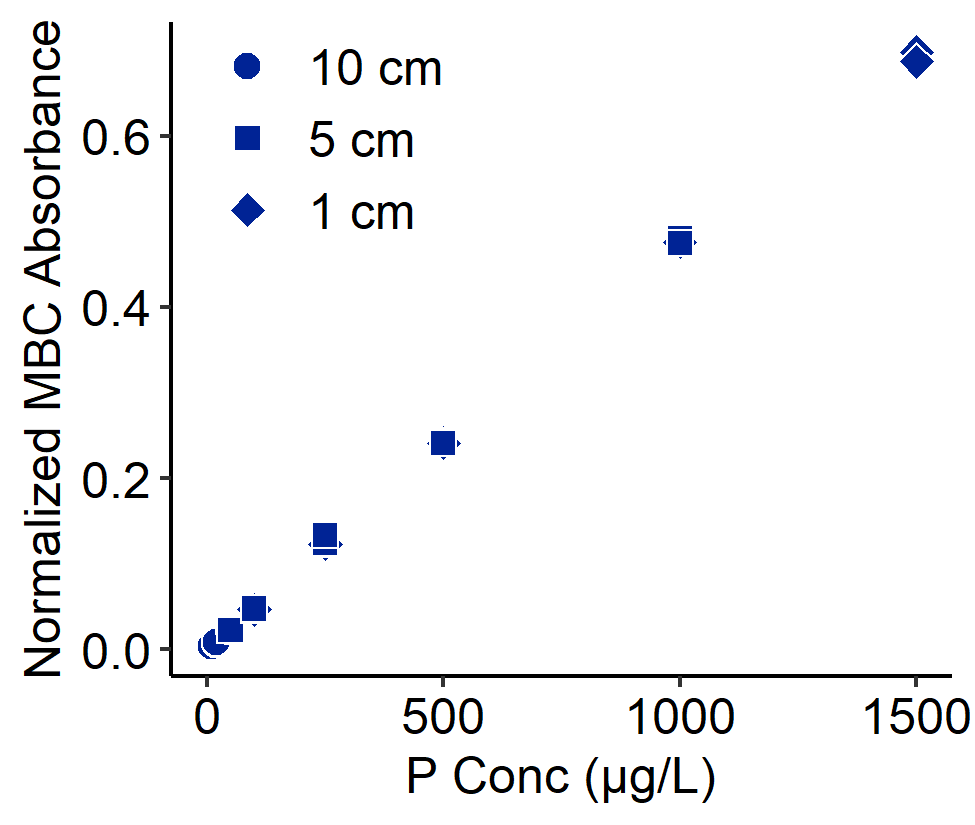

Supplement: S2 Fig — (TIF) [file pone.0317871.s002.tif]

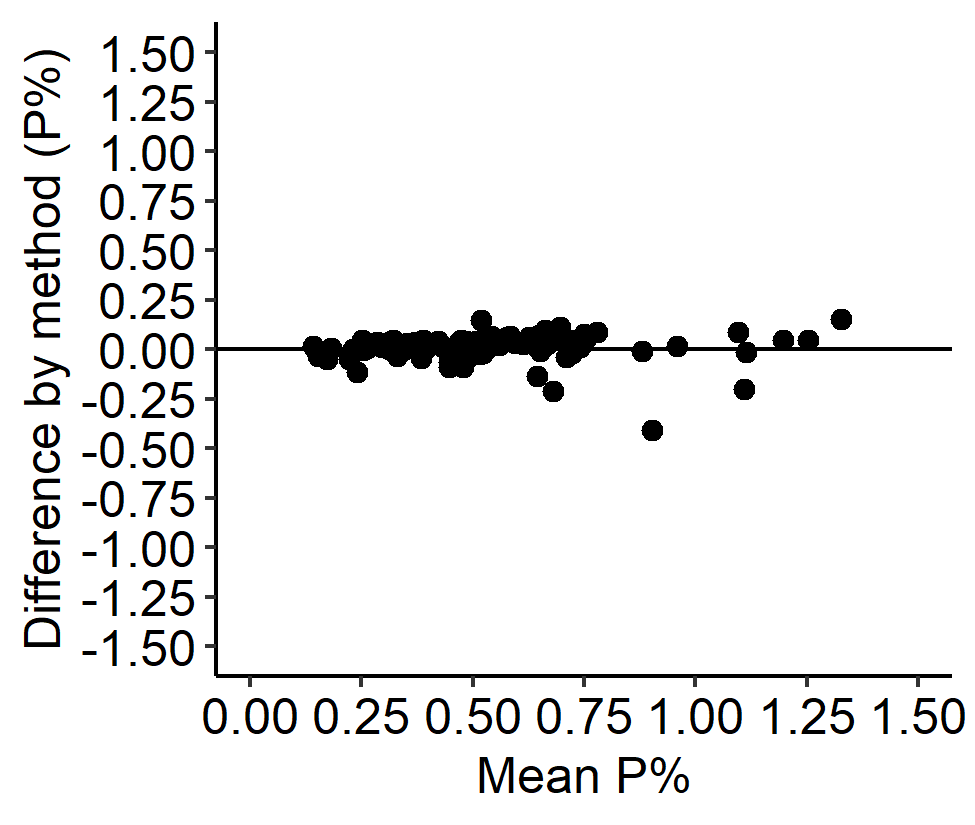

Supplement: S3 Fig — (TIF) [file pone.0317871.s003.tif]
